# Adherence, safety and potential effectiveness of a home‐based Radio‐Taiso exercise program in older adults with frailty: A pilot randomized controlled trial

**DOI:** 10.1111/ggi.14511

**Published:** 2022-11-25

**Authors:** Yosuke Osuka, Hiroyuki Sasai, Narumi Kojima, Masamitsu Sugie, Keiko Motokawa, Kazushi Maruo, Risa Ono, Toshihiko Aoyama, Shigeru Inoue, Hunkyung Kim

**Affiliations:** ^1^ Research Team for Promoting Independence and Mental Health Tokyo Metropolitan Institute of Gerontology Itabashi‐ku Japan; ^2^ Department of Cardiology Tokyo Metropolitan Geriatric Hospital and Institute of Gerontology Itabashi‐ku Japan; ^3^ Department of Biostatistics, Faculty of Medicine University of Tsukuba Tsukuba Japan; ^4^ Japan Radio‐Taiso Federation Chiyoda‐ku Japan; ^5^ Department of Preventive Medicine and Public Health Tokyo Medical University Shinjyuku‐ku Japan

**Keywords:** exercise, feasibility study, frailty, randomized controlled trial, safety

## Abstract

**Aim:**

Radio‐Taiso, a traditional exercise program in Japan, may serve as a coping strategy for older adults with frailty during the COVID‐19 pandemic. This phase II trial tested program adherence and safety and explored the potential effectiveness of a home‐based Radio‐Taiso.

**Methods:**

This assessor‐blind parallel randomized controlled trial included community‐dwelling Jolder Japanese adults with frailty and pre‐frailty. Fifty‐eight eligible participants were randomly allocated to intervention and control groups. Intervention participants performed 3–5‐min sessions of Radio‐Taiso one to four times per day for 12 weeks. Feasibility criteria were set at practice and retention rates of ≥75%. Safety was monitored by assessing all adverse events reported by participants during the intervention period, irrespective of causality. Potential effectiveness was exploratorily assessed using items that allow clinical interpretation of changes: mobility and health‐related quality of life (HR‐QoL), assessed using the modified short physical performance battery (SPPB) and the SF‐36, respectively.

**Results:**

Both practice (83%) and retention rates (100%) met the predetermined feasibility criteria. Eleven adverse events were reported but were supposedly unrelated to the intervention. In the intention‐to‐treat analysis, there was no clinically significant difference in the change in SPPB score between groups (−0.4 points, 95% confidence interval [CI], −1.2, 0.3); however, the intervention group scored higher in the mental component of HR‐QoL than did the control group (3.4 points, 95% CI: −1.1, 7.8).

**Conclusions:**

The preliminary data indicate that a phase III trial is feasible, focusing on the mental aspect of HR‐QoL as the primary outcome. **Geriatr Gerontol Int 2023; 23: 32–37**.

## Graphical abstract

A traditional exercise program called Radio‐Taiso might be a reasonable coping strategy for older Japanese adults with frailty and pre‐frailty during the COVID‐19 pandemic, but no large‐scale clinical trial has evaluated the effectiveness of Radio‐Taiso on clinically important outcomes in this population. This phase II trial tested the adherence, safety and potential effectiveness of a home‐based Radio‐Taiso exercise program, and we examined the feasibility of a phase III trial. Our preliminary data indicated that the phase III trial will be feasible if the mental aspect of health‐related quality of life is used as the primary outcome.

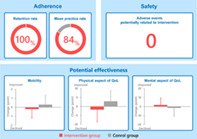

## Introduction

Frailty is a state in which appropriate responses to stressors are weakened because of cumulative decrease in physiological homeostasis among multiple organs.^1^ Fried *et al*.'s frailty phenotype model emphasizes that frailty and pre‐frailty are associated with increased risk of adverse outcomes, including impaired mobility, fall incidence, disability, hospitalization and decline in quality of life (QoL).^2^, ^3^

A nationally representative survey in Japan reported 8.7% and 40.8% prevalence of frailty and pre‐frailty among older adults, respectively.^4^ A sustainable public social insurance system requires intervention programs to improve clinically important outcomes (e.g., mobility, QoL) in older adults with frailty and pre‐frailty. However, to implement such a program in the public healthcare system, its effectiveness should be tested by a clinical trial for individuals with frailty.^1^

A scoping review summarized the effectiveness of 12 randomized controlled trials (RCTs) that screened for frailty before the intervention, among community‐dwelling older adults. The results showed that nine RCTs included exercise interventions that varied by duration, frequency and type of exercises but all effectively reduced frailty scores.^5^ However, these exercise interventions were led by fitness professionals in group sessions attended by participants one to five times per week, implying insufficient evidence on the effectiveness of home‐based exercise programs.^6^ Further, measures to tackle the COVID‐19 pandemic often entail limiting activities outside the home, meaning home‐based exercise programs that older adults with frailty and pre‐frailty can easily practice are essential.

Radio‐Taiso, a traditional exercise program broadcast daily via public radio and television by the Japan Broadcasting Corporation, can be easily accessed/practiced at home by older adults with frailty and pre‐frailty. Our previous studies have identified that participating in calisthenics (as in Radio‐Taiso) reduced risk of decline in cognitive function and instrumental activities of daily living.^7^, ^8^ Thus, Radio‐Taiso may be a reasonable coping strategy for many older Japanese adults with frailty during the COVID‐19 pandemic. A large‐scale clinical trial is needed to evaluate the effectiveness of Radio‐Taiso among older adults with frailty and pre‐frailty; however, the clinically important outcomes that should be focused on remain unknown. Therefore, the impact on various outcomes, including mobility and QoL, should be extensively examined to narrow down the important outcomes that definitive trials focus on. In addition, the safety and feasibility of doing Radio‐Taiso at home, particularly for frail populations that do not habitually practice it, was unclear.

Conducting pilot trials before large‐scale RCTs can help researchers better understand the feasibility of the latter and find potential effectiveness.^9^ To guide the design and hypotheses of a future phase III trial, this phase II trial tested adherence to and safety of Radio‐Taiso, explored its potential effectiveness from multiple perspectives among older adults with frailty and pre‐frailty, and examined the feasibility of a large‐scale RCT.

## Methods

### Design and setting

This study was a randomized, assessor‐blind, parallel‐design, two‐arm, phase II trial, conducted at the Tokyo Metropolitan Institute of Gerontology (TMIG) and in participants' homes nearby. The institutional review board of TMIG reviewed/approved the study protocol. Written informed consent was obtained before the baseline assessment, and patient anonymity was preserved. The protocol was registered in the University Hospital Medical Information Network Clinical Trial Registry on October 12, 2020 (no. UMIN000042083). This study is compliant with the relevant CONSORT 2010 guidelines.^10^

### Recruitment and eligibility criteria

Participants were recruited from the participant pool of a comprehensive geriatric survey conducted by our team once per year. The survey was conducted in October 2020 at the TMIG. Survey respondents who met frailty or pre‐frailty criteria were sent an invitation letter to check their eligibility and invite them to participate.

Inclusion criteria were (i) community‐dwelling older adults aged 65–99 years; (ii) meeting pre‐frailty or frailty criteria according to the revised Japanese Cardiovascular Health Study (see Supporting Information 3 in Appendix S1)^11^; and (iii) providing written informed consent.

Exclusion criteria were (i) being diagnosed with dementia or prescribed anti‐dementia medication; (ii) having a disability in basic activities of daily living; (iii) not being permitted to exercise by a family doctor, except for light‐intensity exercises; (iv) having an unstable medical condition, severe diseases, and not being permitted to participate in the study by a study physician; (v) having a history of angina, myocardial infarction, or heart surgery in the last 3 months, or having received end‐stage disease care or palliative care; (vi) having performed Radio‐Taiso ≥1 day/week in the past month; (vii) having participated in specific rehabilitation programs; (viii) not being able to walk more than 10 m independently; (ix) having participated in other clinical trials; (x) not having a TV; (xi) having difficulty communicating in Japanese; or (xii) having been judged ineligible by a principal investigator/study physician. Moreover, to ensure allocation concealment, participation with housemates was not allowed.

### Randomization and blinding

Participants were randomly allocated to the intervention group (Radio‐Taiso plus nutrition program) or control group (only nutrition program) at a ratio of 1:1. Randomization was performed using computer‐generated blocked randomization (block size = 2) stratified by sex. A principal investigator at the TMIG sent the identification codes for participants to a university biostatistician, who combined prespecified randomization codes with participants' identification codes. Allocation concealment was not applied. Participants and assessors were blinded to group allocation during the trial period, to minimize performance and detection bias. Participants were informed that the nutrition program would be provided to all participants and the exercise program to some participants. They were blinded to the existence of group allocation.

### Intervention

The intervention began on May 17 and ended on August 8, 2021. To address ethical concerns in the control group, all participants were provided the nutrition program. All participants were asked not to change their habitual lifestyle activities during the intervention period.

#### Radio‐Taiso exercise program

Radio‐Taiso includes Radio‐Taiso no. 1 and 2, and Minna no Taiso. These exercises are systematically routinized into eight to 13 rhythmic movements with music. To perform and complete these movements, various physical fitness indices, including endurance, strength, flexibility, coordination and balance, are required; thus, Radio‐Taiso is considered a multicomponent exercise program (see Table 1).

**Table 1:** Characteristics of the Radio‐Taiso program

Name	Time	No. of movements	Contents	URL of videos
Radio‐Taiso no. 1	3 min 10 s	13	Developed to be familiar to all generations from children to older adults at home	https://www.youtube.com/watch?v=SGPBSqxKGAc&t=15s
Radio‐Taiso No. 2	3 min 5 s	13	Exercise intensity is higher than no. 1. Developed to be performed by adults in the workplace	https://www.youtube.com/watch?v=aHlNoTpXf_8
Minna no Taiso	4 min 30 s	8	Exercise intensity is the lowest. Developed to be familiar to all populations, including older adults and people with disabilities	https://www.youtube.com/watch?v=AlNMP0T0B3o&t=8 s

The intervention group was asked to attend three 90‐min in‐person practical sessions provided by certified Radio‐Taiso instructors (the week before the intervention and week 4 and week 8) at the TMIG and to perform Radio‐Taiso at home one to four times per day for 12 weeks. The intervention group was scheduled to participate in a class where they would learn Radio‐Taiso no. 1 and Minna no Taiso movements the week before the intervention and another class to review these movements and learn Radio‐Taiso no. 2 movements at week 4. However, as the fourth wave of COVID‐19 spread to Tokyo, these classes were changed to one‐on‐one instruction via the telephone. The intervention group could also attend a class to review all exercises at week 8.

The exercise program using Radio‐Taiso was stratified by participants' frailty status (pre‐frailty and frailty); a five‐step progressive protocol (level −2 to level +2) was applied (Figure S1 and Supporting Information 1 in Appendix S1). Participants were asked to perform the Radio‐Taiso program while watching TV broadcasts or videos on distributed DVDs. If the participants felt unwell or exercised too intensely, they were asked to stop or reduce the number of exercises. Participants were asked to keep a record of their exercise practice in an exercise diary.

#### Nutrition program

One week before the intervention, a registered dietitian telephoned all participants to inform them how to proceed with the nutrition program and the importance of consuming various foods (Supporting Information 2 in Appendix S1).

### Adherence

Adherence was assessed by practice and retention rates during the intervention period. The practice rate was the number of days Radio‐Taiso was practiced at least once per day, divided by 84 days. The total number of Radio‐Taiso practice sessions over 84 days was also assessed. Feasibility criteria were set at practice and retention rates of ≥75%.^12^

### Safety

The research staff asked participants over telephone whether they had experienced any adverse events, once every 2 weeks. This study defined adverse events as undesired/unintended signs, symptoms, or diseases that occurred during the intervention without clear causality. Adverse events were also objectively evaluated using follow‐up hematological and biochemical tests: white and red blood cell count and aspartate transaminase, alanine transaminase, γ‐glutamyl transpeptidase, platelets, blood urea nitrogen and creatinine levels. These events were assessed as newly identified abnormal values (out of reference range) at week 12. A study physician determined the seriousness of adverse events and their potential association with the intervention.

### Outcomes

This phase II trial was conducted to select the primary outcomes for the phase III trial. Potential effectiveness was exploratorily assessed using surrogate markers that allowed clinical interpretation of changes: mobility was assessed using the modified short physical performance battery (SPPB)^13^, ^14^ and health‐related QoL (HR‐QoL) was assessed using the SF‐36. The SPPB is a reliable and valid surrogate marker of frailty and mobility changes.^15^ Previous research provides a detailed assessment of the SPPB.^13^ Scores range from 0 to 10, with a higher score indicating better mobility. A difference of ≥1.0 point between the two groups has been recommended as the minimal clinically important difference (MCID).^16^ HR‐QoL was evaluated using the Japanese version of the SF‐36, a widely used, reliable, and valid tool.^17^, ^18^ The physical component summary (PCS) and mental component summary (MCS) scores were used as outcomes,^19^ standardized as T‐scores using the 2017 Japanese national standard values.^20^ A difference between groups of ≥2.0 points for PCS and ≥3.0 points for MCS has been recommended as the MCID.^21^

Other outcomes, including frailty phenotype score, motor and cognitive function, body composition, exercise self‐efficacy, depressive mood, social network, functional capacity, habitual energy intake, physical activity, sleep condition and blood parameters were also assessed to explore potential effectiveness from multiple perspectives (see Supporting Information 3 in Appendix S1).

### Sample size

We hypothesized that the Radio‐Taiso intervention would yield small/medium standardized effect sizes (Cohen's *d* = 0.2–0.6) in the phase III trial. A pilot study (*n* ≥ 55) offered the minimum overall sample size for these effect sizes based on the applied upper confidence limit approach.^22^ Considering a drop‐out rate of approximately 10% after allocation, we recruited 60 participants.

### Statistical analysis

To examine the potential effectiveness of Radio‐Taiso, between‐group differences in outcome changes were estimated at 95% confidence interval. In addition, effect sizes for between‐group differences in outcome changes were calculated. Change in value was calculated by subtracting the follow‐up value from the baseline value. Missing data were treated using the list‐wise deletion method. Based on the intention‐to‐treat principle, a full analysis set with complete baseline and follow‐up survey data was used in the main analysis.^23^ All analyses were performed using R version 4.1.2 (R Foundation, Vienna, Austria).

## Results

### Enrollment

**Figure 1:**
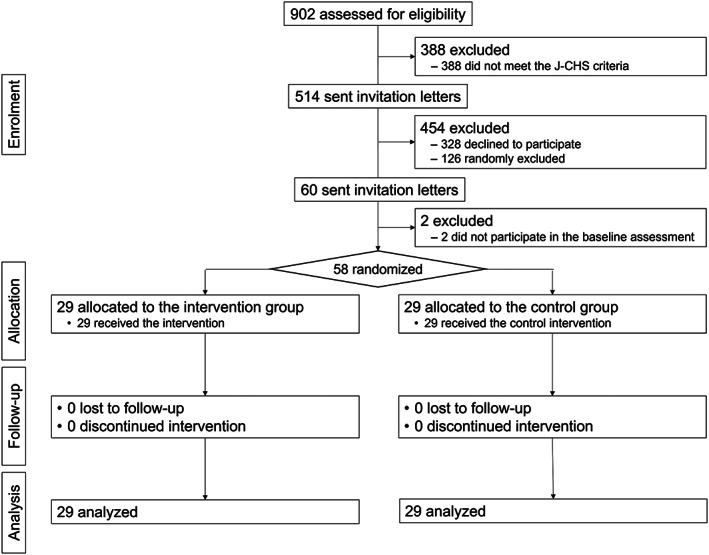
Flow diagram of the study.

Figure 1 shows the study flow. During October 2020, 902 older adults participated in the comprehensive geriatric survey. Of these, 514 participants who met the pre‐frailty or frailty criteria received the invitation letter. In total, 186 individuals who met all the eligibility criteria volunteered to participate, among which 60 were randomly selected and sent a baseline assessment invitation. Fifty‐eight older adults participated in the baseline assessment and were randomly assigned to two groups. No participants withdrew from the intervention or were lost to follow‐up.

### Baseline characteristics

Table 2 shows the baseline characteristics of participants. The median age was 77 years (interquartile range, 73–80). Overall, 84.5% were women; 10.3% were older adults with frailty; and the median SPPB score at baseline assessment for all participants was 6 points (interquartile range, 5–8).

**Table 2:** Characteristics of the study participants

Characteristics	Intervention group	Control group
	*N* = 29	*N* = 29
Age, years	77 [72, 82]	77 [74, 79]
Sex, women	25 (86.2)	24 (82.8)
Hypertension, yes	16 (55.2)	7 (24.1)
Heart disease, yes	9 (31.0)	2 (6.9)
Diabetes, yes	8 (27.6)	4 (13.8)
Hyperlipidemia, yes	13 (44.8)	8 (27.6)
Osteoporosis, yes	9 (31.0)	9 (31.0)
Respiratory disease, yes	2 (6.9)	4 (13.8)
Low‐back pain, yes	10 (34.5)	8 (27.6)
Knee pain, yes	9 (31.0)	10 (34.5)

Data are shown as median [interquartile range] or *n* (%).

### Adherence outcomes

All participants in both groups completed the follow‐up assessment (retention rate, 100%). Exercise and nutrition diaries were collected from all participants. Of the exercise diaries returned, the mean practice rate of Radio‐Taiso was 83.8 ± 30.0%, and the mean total number of Radio‐Taiso practices during the intervention period was 222.2 ± 94.4. Four participants in the intervention group did not meet the total adherence rate of 75%. Two participants fell and were injured before the intervention and were told to stop performing Radio‐Taiso by their family doctors.

### Adverse outcomes

Table 3 shows the details of the adverse events reported by participants during the intervention period and the abnormal blood parameter values in the follow‐up assessment. In total, 11 adverse events were reported by intervention participants, and 29 abnormal values in blood parameters were observed at week 12; however, a study physician declared them unrelated to the intervention.

**Table 3:** Adverse events during the intervention period

Outcomes	Intervention group	Control group
	*n* = 29	*n* = 29
Adverse events reported by participants		
All	11	3
Pain	5	2
Fall	3	0
Side effects potentially related to COVID‐19 vaccines	2	1
Shingles	1	0
Adverse event assessed by abnormal values in the blood test^†^		
All	29	26
WBC, ref. (M: 3.9–9.8 10^3^/μL, F: 3.5–9.1 10^3^/μL)	3	2
RBC, ref. (M: 427–570 10^4^/μL, F: 376–500 10^4^/μL)	2	0
AST, ref. (0–40 U/L)	2	1
ALT, ref. (0–45 U/L)	4	1
γ‐GTP, ref. (M: 0–75 U/L, F: 0–45 U/L)	5	2
Platelet, ref. (13–36.9 10^4^/μL)	1	1
Blood urea nitrogen, ref. (8–22 mg/dL)	6	9
Creatine, ref. (M: 0.61–1.07 mg/dL, F: 0.47–0.79 mg/dL)	6	10

Data shown as *n*. ^†^Newly identified signs (out of the reference range) at week 12. ALT, alanine transaminase; AST, aspartate aminotransferase; γ‐GTP, γ‐glutamyl trans peptidase; RBC, red blood cell; WBC, white blood cell.

### Potential effectiveness outcome

Table 4 shows results only for outcomes that can indicate clinically important changes. Although the difference in mean change was not significant, the SPPB score in the control group was slightly increased, whereas that in the intervention group was slightly decreased. Improvement in the PCS score in the control group was significantly greater than in the intervention group. In contrast, the MCS score was slightly increased in the intervention group and slightly decreased in the control group. Results for other outcomes are presented in Table S1.

**Table 4:** Mean differences in the outcomes between the intervention and control groups

Outcomes	Intervention group, *N* = 29			Control group, *N* = 29				
	Baseline	Follow‐up	Change	Baseline	Follow‐up	Change	Group difference	Cohen's *d*
SPPB, point	6.2 ± 1.9	6.0 ± 2.0	−0.2 ± 1.6	6.7 ± 2.1	7.0 ± 1.8	0.3 ± 1.0	−0.4 (−1.2, 0.3)	0.337
PCS, point	43.9 ± 9.8	42.1 ± 9.8	−1.8 ± 6.7	44.8 ± 7.0	47.8 ± 8.9	2.9 ± 5.9	−4.8 (−8.1, −1.4)	0.755
MCS, point	53.8 ± 8.4	55.7 ± 7.2	1.9 ± 7.7	57.0 ± 9.1	55.5 ± 8.1	−1.5 ± 9.2	3.4 (−1.1, 7.8)	0.395

Baseline, follow‐up and change values are shown as mean ± SD. Group difference is shown as mean (95% confidence interval). MCS, mental component summary; PCS, physical component summary; SPPB, short physical performance battery.

## Discussion

The success of a pilot trial does not necessarily require the detection of the significant effectiveness but instead provides sufficient evidence to determine the possibility of proceeding to the main trial.^9^ Both practice (83%) and retention rates (100%) met the predetermined feasibility criteria, and no adverse events potentially related to intervention were observed. Compared with the control group, the intervention group showed no trend of clinically important changes in physical outcomes, including mobility and PCS score, but did show a clinically important improvement in MCS. These preliminary data indicate that the phase III trial will be feasible if the mental aspect of HR‐QoL is used as the primary outcome.

The retention rate in this study was much better than that reported in the results of a systematic review on home‐based exercise programs.^24^ The overall practice rate averaged approximately 83%, which exceeded the predetermined success criterion (75%).^12^ This good adherence may be because Radio‐Taiso is familiar to many older Japanese adults, helping them readily incorporate it into their daily lives. Furthermore, the mean total number of Radio‐Taiso practice sessions completed per participant during the intervention period was approximately 222, or an average of 2.6 times per day. Thus, it would be possible for the target population to perform Radio‐Taiso two to three times daily in the main trial.

The difference in the change in SPPB scores between the two groups was <1.0 point, which is the value that configures an MCID,^16^ indicating that this preliminary estimation cannot be considered a clinically important change in mobility. In contrast, the control group showed a change of ≥2 points in PCS score compared with the intervention group, a higher difference than for the MCID.^21^ No clinically important changes were observed in the other outcomes, including motor and cognitive function (Table S1).

The reasons for the lack of positive results in these outcomes are unclear. We revised and reported the protocol for the phase III trial to address methodological concerns.^25^ For example, we will increase face‐to‐face instruction from three to six sessions, as several participants commented that “there is a difference in consideration for the quality of practice between imitating a movement on TV or DVD and understanding the purpose of a movement through face‐to‐face instruction.” In addition, the phase III trial will introduce a daily exercise calendar. Participants could learn the effective and safe practice of Radio‐Taiso and reflect on whether they had complied with it daily. Addressing these concerns will enhance the feasibility and scientific validity of the phase III trial.

The difference in the MCS score between the two groups was >3.0 points that configure an MCID.^21^ An observational study showed that exercising during the COVID‐19 pandemic positively affected the mental health of older adults.^26^ Therefore, our preliminary estimates may provide the data needed for the design of a future phase III trial aimed at identifying the effectiveness of Radio‐Taiso on MCS (as the primary outcome).

This phase II trial could not determine whether exercise adherence and effectiveness differed by participant characteristics (e.g., sex, age, frailty severity), owing to the small sample size. Thus, we cannot discuss the generalizability of its feasibility and efficacy; accordingly, the phase III trial will apply subgroup analyses and assess heterogeneity by using a larger sample size.^25^ Furthermore, although the assessors were blinded to the allocation, we could not objectively assess whether the blinding was successful; accordingly, the phase III trial should confirm the effectiveness of the blinding of assessors.

## Conclusion

This phase II trial did not show a clinically important difference in physical outcomes between the intervention and control groups. However, the intervention group exhibited a trend toward a clinically important improvement in the mental aspects of HR‐QoL compared with the control group. Furthermore, program adherence was good, and adverse events related to the intervention were not observed. These preliminary data indicate that a phase III trial focusing on the mental aspect of HR‐QoL (as the primary outcome) is feasible.

## Disclosure statement

YO served as a principal investigator for the collaborative research agreement between TMIG and Japan Post Insurance Co., Ltd. RO and TA are commissioned as certified instructors of the Japan Radio‐Taiso Federation. These organizations, Japan Post Insurance Co., Ltd. and the Japan Radio‐Taiso Federation, hold a mission to disseminate and promote Radio‐Taiso. The other authors declare no conflict of interest.

## Supporting information


**Table S1.** Mean differences in the other outcomes between the intervention and control groups.

**Figure S1.** Protocol of the home‐based Radio‐Taiso exercise program for community‐dwelling Japanese older adults with frailty and pre‐frailty.

**Appendix S1.** Supporting Information.

## Data Availability

Research data are not shared.
