# Supplementary material for: Adherence, safety and potential effectiveness of a home‐based Radio‐Taiso exercise program in older adults with frailty: A pilot randomized controlled trial
Source: Geriatr Gerontol Int. 2022 Nov 25;23(1):32–7. doi: 10.1111/ggi.14511 (PMC10100027; doi:10.1111/ggi.14511)
Supplement: Supplementary file 1 — Table S1. Mean differences in the other outcomes between the intervention and control groups. [file GGI-23-32-s002.docx]

| **Table S1.** **Mean differences in the other outcomes between the intervention and control groups** | | | | | | | | | | | |
| --- | --- | --- | --- | --- | --- | --- | --- | --- | --- | --- | --- |
|  |  | Intervention group, n = 29 | | |  | Control group, n = 29 | | |  |  |  |
|  |  | Baseline | Follow-up | Change |  | Baseline | Follow-up | Change |  | Group difference | Cohen's *d* |
| **Frailty status** |  |  |  |  |  |  |  |  |  |  |  |
| Frailty phenotype, point |  | 1.4 (0.8) | 0.9 (0.6) | -0.6 (0.7) |  | 1.4 (0.8) | 0.8 (0.7) | -0.6 (0.8) |  | 0.0 (-0.4, 0.4) | N.A. |
| **Motor function** |  |  |  |  |  |  |  |  |  |  |  |
| Gait speed, cm/s |  | 115.5 (17.9) | 121.9 (22.1) | 6.4 (11.1) |  | 125.6 (22.3) | 131.2 (21.3) | 5.7 (8.7) |  | 0.7 (-4.5, 6.0) | 0.073 |
| Right stride length, cm |  | 119.1 (14.5) | 60.4 (8.7) | 2.5 (7.6) |  | 127.6 (15.9) | 64.9 (8.7) | 2.9 (6.2) |  | -0.4 (-4.1, 3.2) | 0.061 |
| Left stride length, cm |  | 119.0 (14.5) | 60.6 (9.2) | 2.3 (7.4) |  | 127.5 (16.1) | 65.1 (8.0) | 2.8 (6.3) |  | -0.5 (-4.1, 3.1) | 0.073 |
| Sit and reach, cm |  | 35.3 (7.3) | 35.6 (8.0) | 0.3 (4.1) |  | 36.1 (11.2) | 36.5 (12.6) | 0.4 (5.3) |  | -0.1 (-2.6, 2.4) | 0.022 |
| Timed up-and-go, s |  | 9.7 (1.4) | 8.7 (1.8) | 0.0 (0.8) |  | 8.3 (1.7) | 8.2 (1.6) | -0.1 (0.8) |  | 0.0 (-0.4, 0.5) | 0.056 |
| KES, newton |  | 231.3 (70.6) | 214.8 (70.5) | -18.7 (34.0) |  | 239.7 (59.1) | 230.3 (59.1) | -9.3 (45.4) |  | -9.4 (-30.9, 12.0) | 0.235 |
| **Body composition** |  |  |  |  |  |  |  |  |  |  |  |
| Body fat percent, % |  | 30.2 (7.6) | 32.6 (7.9) | 2.3 (6.6) |  | 27.7 (6.2) | 27.2 (8.4) | -0.5 (9.9) |  | 2.8 (-1.6, 7.3) | 0.337 |
| Fat free mass, kg |  | 39.5 (8.2) | 38.1 (7.1) | -1.4 (7.5) |  | 40.4 (7.9) | 38.6 (6.3) | -1.8 (8.7) |  | 0.4 (-3.8, 4.7) | 0.053 |
| **Cognitive function** |  |  |  |  |  |  |  |  |  |  |  |
| SDST, score |  | 47.9 (7.0) | 49.0 (8.6) | 1.2 (9.3) |  | 46.5 (8.5) | 48.2 (9.1) | 1.7 (7.6) |  | -0.5 (-4.9, 4.0) | 0.057 |
| Verbal memory, score |  | 8.6 (1.1) | 8.5 (0.9) | -0.1 (1.3) |  | 7.9 (1.6) | 8.4 (1.2) | 0.5 (1.3) |  | -0.6 (-1.3, 0.1) | 0.472 |
| Logical memory, score |  | 8.7 (1.2) | 8.6 (1.5) | -0.1 (1.8) |  | 7.5 (2.5) | 8.2 (1.9) | 0.7 (2.5) |  | -0.9 (-2.0, 0.3) | 0.393 |
| Trail Making Test–A, s |  | 20.3 (4.3) | 19.9 (5.3) | -0.4 (6.4) |  | 20.6 (8.5) | 21.6 (9.1) | 1.0 (11.1) |  | -1.4 (-6.2, 3.3) | 0.160 |
| Trail Making Test–B, s |  | 39.3 (33.8) | 40.9 (18.0) | 1.6 (36.4) |  | 39.5 (21.7) | 37.9 (19.5) | -1.6 (20.4) |  | 3.1 (-12.4, 18.6) | 0.106 |
| **Exercise self-efficacy** |  |  |  |  |  |  |  |  |  |  |  |
| HEBS, point |  | 20.2 (4.8) | 20.5 (5.1) | 0.3 (5.0) |  | 19.5 (5.1) | 19.3 (5.0) | -0.2 (4.9) |  | 0.5 (-2.1, 3.1) | 0.104 |
| **Depressive mood** |  |  |  |  |  |  |  |  |  |  |  |
| GDS, point |  | 2.5 (2.0) | 2.3 (2.0) | -0.1 (1.5) |  | 3.6 (3.0) | 3.8 (3.5) | 0.3 (2.4) |  | -0.4 (-1.5, 0.6) | 0.210 |
| **Social support** |  |  |  |  |  |  |  |  |  |  |  |
| LSNS-6, point |  | 16.5 (4.2) | 16.6 (4.0) | 0.1 (3.6) |  | 12.6 (5.6) | 11.6 (5.8) | -1.0 (3.9) |  | 1.1 (-0.8, 3.1) | 0.302 |
| **Functional capacity** |  |  |  |  |  |  |  |  |  |  |  |
| Kihon check list, point |  | 5.0 (3.0) | 4.6 (3.0) | -0.4 (2.2) |  | 5.0 (3.3) | 5.5 (4.0) | 0.5 (2.7) |  | -0.9 (-2.2, 0.4) | 0.379 |
| **Dietary intake** |  |  |  |  |  |  |  |  |  |  |  |
| Energy intake, kcal/day |  | 1936 (606) | 1954 (621) | 18 (537) |  | 2008 (561) | 1930 (498) | -78(476) |  | 95.7 (-171, 362) | 0.189 |
| **Physical activity** |  |  |  |  |  |  |  |  |  |  |  |
| Step counts, steps/day |  | 4760 (3853) | 3823 (2415) | -937 (2482) |  | 4369 (2863) | 3708 (2746) | -661 (1100) |  | -275.9 (-1286, 734) | 0.144 |
| **Sleep condition** |  |  |  |  |  |  |  |  |  |  |  |
| Sleep efficacy, % |  | 86.3 (5.3) | 82.0 (7.2) | -4.4 (4.7) |  | 87.3 (3.9) | 83.6 (5.8) | -3.4 (3.7) |  | -1.0 (-3.2, 1.3) | 0.226 |
| PSQI, point |  | 6.2 (3.2) | 7.1 (3.4) | 0.9 (2.4) |  | 6.4 (3.1) | 6.9 (3.9) | 0.5 (2.7) |  | 0.4 (-1.0, 1.7) | 0.150 |
| **Blood test** |  |  |  |  |  |  |  |  |  |  |  |
| Hemoglobin, g/dL |  | 13.5 (1.3) | 13.2 (1.2) | -0.3 (0.5) |  | 13.7 (1.0) | 13.4 (1.1) | -0.3 (0.5) |  | 0.0 (-0.3, 0.2) | 0.014 |
| Hematocrit, % |  | 43.0 (3.5) | 42.7 (3.7) | -0.2 (1.5) |  | 43.7 (3.1) | 43.5 (3.2) | -0.2 (1.7) |  | 0.0 (-0.9, 0.8) | 0.027 |
| Blood glucose, mg/dL |  | 114.3 (33.5) | 116.4 (32.7) | 2.1 (21.1) |  | 111.8 (28.8) | 112.8 (32.9) | 1.0 (16.6) |  | 1.2 (-8.8, 11.2) | 0.062 |
| Total protein |  | 7.2 (0.3) | 7.1 (0.3) | -0.1 (0.2) |  | 7.2 (0.4) | 7.2 (0.5) | -0.1 (0.3) |  | -0.1 (-0.2, 0.1) | 0.194 |
| Albumin |  | 4.5 (0.2) | 4.3 (0.2) | -0.2 (0.2) |  | 4.5 (0.2) | 4.3 (0.3) | -0.2 (0.2) |  | -0.1 (-0.2, 0.1) | 0.264 |
| LDH, IU/L |  | 225.4 (40.7) | 230.5 (31.0) | 5.0 (35.4) |  | 207.7 (36.7) | 217.8 (34.0) | 10.0 (18.4) |  | -5.0 (-19.8, 9.8) | 0.178 |
| Fe, µg/dL |  | 105.9 (29.7) | 104.6 (27.4) | -1.4 (29.4) |  | 95.9 (25.1) | 101.9 (23.2) | 6.0 (23.3) |  | -7.4 (-21.4, 6.5) | 0.279 |
| Total cholesterol, mg/dL |  | 218.4 (29.2) | 212.0 (29.9) | -6.4 (16.1) |  | 236.2 (37.4) | 228.6 (37.9) | -7.7 (12.1) |  | 1.2 (-6.3, 8.7) | 0.085 |
| LDLC, mg/dL |  | 119.0 (25.9) | 112.9 (23.6) | -6.1 (13.5) |  | 131.1 (31.2) | 122.9 (32.3) | -8.2 (10.5) |  | 2.1 (-4.3, 8.4) | 0.171 |
| HDLC, mg/dL |  | 72.4 (19.0) | 65.3 (16.4) | -7.1 (5.8) |  | 73.9 (23.6) | 67.2 (21.5) | -6.7 (7.4) |  | -0.4 (-3.9, 3.1) | 0.057 |
| Triglyceride, mg/dL |  | 120.4 (58.2) | 134.9 (76.6) | 14.5 (51.9) |  | 148.1 (68.7) | 164.9 (99.3) | 16.8 (83.7) |  | -2.3 (-38.9, 34.3) | 0.033 |
| H-CRP, mg/dL |  | 0.07 (0.08) | 0.1 (0.3) | 0.07 (0.25) |  | 0.09 (0.17) | 0.1 (0.1) | -0.02 (0.15) |  | 0.1 (0.0, 0.2) | 0.436 |
| Baseline, follow-up, and change values shown as mean (standard deviation). Group difference shown as mean (95% confidence interval). KES, knee extensor strength; SDST, Symbol Digit Substitution Task; HEBS, Home-Exercise Barrier Self-Efficacy Scale; GDS, Geriatric Depression Scale; LSNS-6, Lubben Social Network Scale-6; PSQI, Pittsburgh Sleep Quality Index; LDH, lactate dehydrogenase; LDLC, low-density lipoprotein cholesterol; HDLC, high-density lipoprotein cholesterol; H-CRP, high-sensitivity C-reactive protein; N.A., not applicable.  There was one missing data on sleep efficacy at baseline, but no missing data at follow-up. There was one missing data on knee extensor strength at baseline and one at follow-up. There were no missing data for other items. | | | | | | | | | | | |
