# Supplementary material for: Adherence, safety and potential effectiveness of a home‐based Radio‐Taiso exercise program in older adults with frailty: A pilot randomized controlled trial
Source: Geriatr Gerontol Int. 2022 Nov 25;23(1):32–7. doi: 10.1111/ggi.14511 (PMC10100027; doi:10.1111/ggi.14511)
Supplement: Supplementary file 2 — Figure S1. Protocol of the home‐based Radio‐Taiso exercise program for community‐dwelling Japanese older adults with frailty and pre‐frailty. [file GGI-23-32-s001.pptx]

## Slide 1
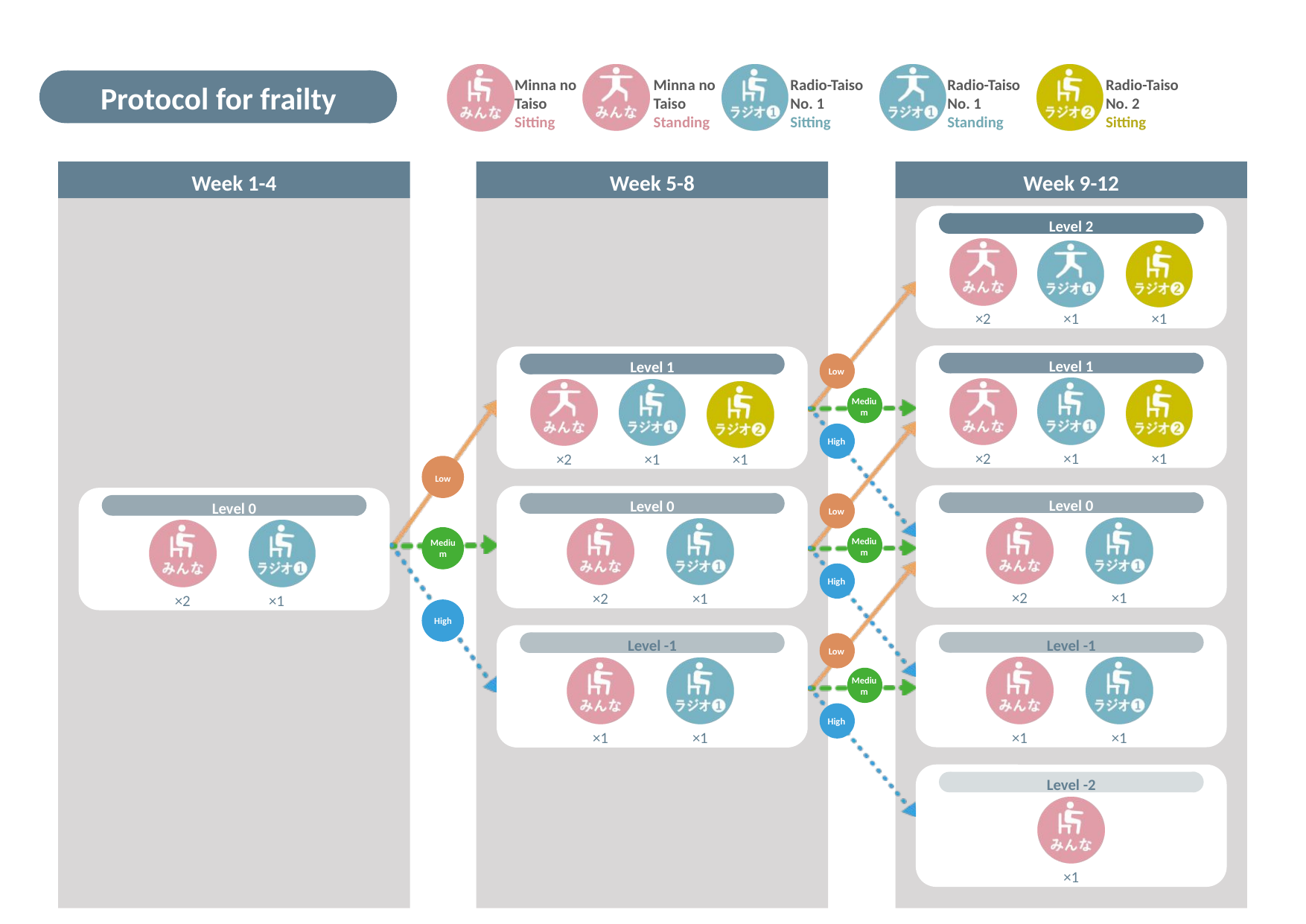

Minna no
Taiso
Standing
Radio-Taiso
No. 1
Sitting
Radio-Taiso
No. 1
Standing
Radio-Taiso
No. 2
Sitting
Minna no
Taiso
Sitting
Protocol for frailty
Week 1-4
Week 5-8
Week 9-12
Level 2
×2
×1
×1
Low
Medium
High
Level 1
×1
×2
×1
Level 1
×1
×2
×1
Low
Medium
High
Low
Level 0
×2
×1
Level 0
×2
×1
Level 0
×2
×1
Medium
Low
Medium
High
High
Level -1
×1
×1
Level -1
×1
×1
Level -2
×1

## Slide 2
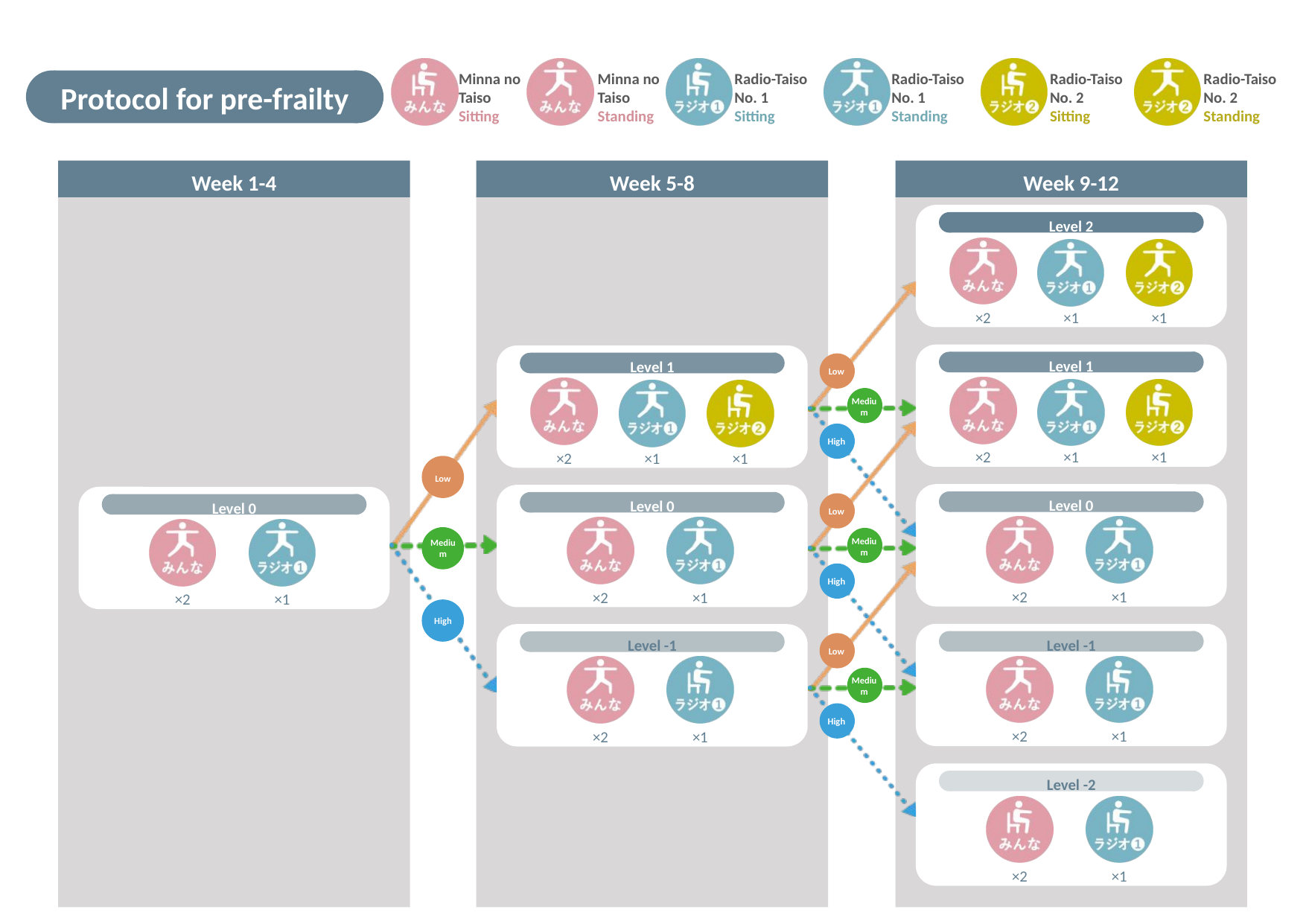

Minna no
Taiso
Standing
Radio-Taiso
No. 1
Sitting
Radio-Taiso
No. 1
Standing
Radio-Taiso
No. 2
Sitting
Radio-Taiso
No. 2
Standing
Minna no
Taiso
Sitting
Protocol for pre-frailty
Week 1-4
Week 5-8
Week 9-12
×2
×1
×1
Level 2
Low
Medium
High
×2
×1
×1
×2
×1
×1
Level 1
Level 1
Low
Medium
High
Low
×2
×1
×2
×1
×2
×1
Level 0
Level 0
Level 0
Medium
Low
Medium
High
High
×2
×1
×2
×1
Level -1
Level -1
Level -2
×2
×1
