# Supplementary material for: Adherence, safety and potential effectiveness of a home‐based Radio‐Taiso exercise program in older adults with frailty: A pilot randomized controlled trial
Source: Geriatr Gerontol Int. 2022 Nov 25;23(1):32–7. doi: 10.1111/ggi.14511 (PMC10100027; doi:10.1111/ggi.14511)
Supplement: Supplementary file 3 — Appendix S1. Supporting Information. [file GGI-23-32-s003.docx]

# Supporting Information 1

The rating of perceived exertion (RPE) of participants was assessed at weeks 4 and 8 using Borg’s RPE scale, and the exercise load was adjusted by using each participant’s most recent RPE, as follows: 1) the load was increased by one level if the RPE score was in the range of 6–11 (based on Borg’s RPE scale, this range implies that the exercise is perceived as of low intensity); 2) the load was maintained if the RPE score was in the range of 12–16 (medium intensity); and 3) the load was decreased by one level if the RPE score was in the range of 17–20 (high intensity).

# Supporting Information 2

The nutrition program comprised telephone consultations and the distribution of nutritional leaflets and diaries. The leaflets were distributed once a week during the first four weeks from the start of the intervention, and they detailed the roles of protein, calcium, vitamins/minerals, and carbohydrates/lipids, and provided information on the recommended servings of different food items, as well as specific recipes, to ensure the intake of these nutrients. Participants were asked to record whether they consumed each of the 10 food groups in a nutrition diary using a dietary variety score^27^. They could consult a registered dietitian one day per week by telephone.

# Supporting Information 3

### Frailty phenotype score

The frailty phenotype score was assessed using the Japanese version of the Cardiovascular Health Study criteria, which evaluates weight loss, weakness, slowness, exhaustion, and low activity. The pre-frailty and frailty statuses were defined if the person had 1–2 and ≥ 3 limitations in these five parameters, respectively^28^. A detailed assessment of each condition has been described in our previous study^29^

### Motor and cognitive function

Usual gait performance, including gait speed (cm/s) and stride length (cm), was assessed using an electronic walkway device (GAITRite Systems, CIR Systems, Inc., NJ, USA). Dynamic balance was assessed using Timed Up and Go test. The extensibility of the hamstrings was assessed using a sit-and-reach test with a digital device (T.K.K.5412; Takei Scientific Instruments Co., Ltd., Niigata, Japan). Isometric knee extensor strength was measured using a handheld dynamometer (µTas F-1; Anima, Tokyo, Japan). The assessment methods for all these tests have been detailed in previous studies^30-33^.

The computerized multidimensional neurocognitive task battery was used to assess cognitive function. This included information processing, which was assessed by the Symbol Digit Substitution Task; immediate recognition, which was assessed by verbal memory and logical memory test; attention, which was assessed by the Trail Making Test, Part A; and executive function, which was assessed by Trail Making Test, Part B. The detailed assessment and scoring methods for all these cognition-related tests have been described previously^34^.

### Body composition

Body fat percentage and fat-free mass were quantified using a validated segmental multi-frequency bioelectrical impedance analysis (InBody 720; Biospace, Seoul, Korea)^35^.

### Exercise self-efficacy

The Home-exercise Barrier Self-efficacy Scale was used to assess participants’ confidence in performing exercises at home^36^. Participants were asked to answer to six items on different exercise situations at home on a five-point Likert scale, ranging from 1 (*not confident at all*) to 5 (*absolutely confident*). The scores range from 5–30, with higher scores indicating greater exercise self-efficacy. Detailed descriptions of the questions and the scale can be found in a prior study^36^.

### Depressive mood

Depressive mood was assessed using a short version of the Geriatric Depression Scale^37^. Participants answered “yes” or “no” to 15 questions about their daily mood. Scores range from 0–15, with higher scores indicating a more severely depressed mood. Detailed descriptions of the questions and the scale can be found in a prior study^37^.

### Social network

Social networks were evaluated using the Japanese version of the Lubben Social Network Scale-6^38^. It comprises six questions about emotional and instrumental support from family and non-family members. The score ranges from 0–30 points, with a higher score indicating a larger social network. Detailed descriptions of the questions and the scale can be found in a prior study^38^.

### Functional capacity

Functional capacity was evaluated using the Kihon Checklist, which assesses all aspects of frailty, including instrumental activities of daily living, motor function, nutrition, oral function, cognitive function, social function, and depressed mood. The score ranges from 0–25 points, with higher scores indicating lower functional capacity^39^. Detailed descriptions of the questions and the scale can be found in a prior study^39^.

### Habitual dietary intake, physical activity, sleep condition, and sleep quality

Habitual dietary intake per day (kcal/day) for the preceding month was measured using a brief self-administered diet history questionnaire^40,41^. Habitual physical activity and sleep conditions were assessed by the number of steps per day and sleep efficiency (i.e., the proportion of the time sleeping during the time spent in bed), respectively. We used two validated tri-axis accelerometers and algorithms to collect the related data (Active style Pro HJA-750C; Omron Healthcare, Tokyo, Japan, ActiGraph GT3X +; ActiGraph, FL, USA)^42,43^. These devices were worn on the hip and non-dominant wrist for seven days after the baseline and the follow-up assessments. Records were defined as valid when the device was worn for at least 10 hours a day on the hip and each wrist^44^. If a participant provided valid records for three days or more, the number of steps and sleep efficiency were calculated for that participant. Subjective sleep quality was assessed using the Japanese version of the Pittsburgh Sleep Quality Index^45,46^.

### Blood test

A nurse obtained a non-fasting blood sample from each participant’s anterior elbow vein. Blood parameters for assessing exploratory efficacy included hemoglobin, hematocrit, blood glucose, total protein, albumin, lactate dehydrogenase, iron, total cholesterol, low-density lipoprotein cholesterol, high-density lipoprotein cholesterol, triglyceride, and high-sensitivity C-reactive protein. Venous blood was centrally analyzed by an independent research institute (Health Sciences Research Institute, Kanagawa, Japan). Detailed descriptions of the analysis methods can be found in a previous study^47^.

# Supporting Information References

27. Kumagai S, Watanabe S, Shibata H *et al.* [Effects of dietary variety on declines in high-level functional capacity in elderly people living in a community]. *Nihon Koshu Eisei Zasshi* 2003; 50: 1117-1124.

28. Satake S, Shimada H, Yamada M *et al.* Prevalence of frailty among community-dwellers and outpatients in Japan as defined by the Japanese version of the Cardiovascular Health Study criteria. *Geriatrics & Gerontology International* 2017; 17: 2629-2634.

29. Osuka Y, Kojima N, Yoshida Y *et al.* Exercise and/or dietary varieties and incidence of frailty in community-dwelling older women: a 2-year cohort study. *The Journal of Nutrition, Health & Aging* 2019; 23: 425-430.

30. Montero-Odasso M, Casas A, Hansen KT *et al.* Quantitative gait analysis under dual-task in older people with mild cognitive impairment: a reliability study. *Journal of Neuroengineering and Rehabilitation* 2009; 6: 35.

31. Podsiadlo D, Richardson S. The timed "Up & Go": a test of basic functional mobility for frail elderly persons. *Journal of the American Geriatrics Society* 1991; 39: 142-148.

32. Suwa M, Imoto T, Kida A, Yokochi T, Iwase M, Kozawa K. Association of body flexibility and carotid atherosclerosis in Japanese middle-aged men: a cross-sectional study. *BMJ Open* 2018; 8: e019370.

33. Kojima N, Kim H, Saito K *et al.* Association of knee-extension strength with instrumental activities of daily living in community-dwelling older adults. *Geriatrics & Gerontology International* 2014; 14: 674-680.

34. Makizako H, Shimada H, Park H *et al.* Evaluation of multidimensional neurocognitive function using a tablet personal computer: test-retest reliability and validity in community-dwelling older adults. *Geriatrics & Gerontology International* 2013; 13: 860-866.

35. Kim M, Kim H. Accuracy of segmental multi-frequency bioelectrical impedance analysis for assessing whole-body and appendicular fat mass and lean soft tissue mass in frail women aged 75 years and older. *European Journal of Clinical Nutrition* 2013; 67: 395-400.

36. Arita N, Takenaka K, Shimazaki T. Development of a home-exercise barrier self-efficacy scale for elderly people requiring support and care. *Journal of the Japanese Physical Therapy Association* 2014; 41: 338-346.

37. Yatomi N. The factor structure and item characteristic of the GDS (Geratric Depression Scale) short version in a Japanese elderly sample. *Japanese Journal of Gerontology* 1994; 16: 29-36.

38. Kurimoto A, Awata S, Ohkubo T *et al.* [Reliability and validity of the Japanese version of the abbreviated Lubben Social Network Scale]. *Nihon Ronen Igakkai Zasshi* 2011; 48: 149-157.

39. Sewo Sampaio PY, Sampaio RA, Yamada M, Arai H. Systematic review of the Kihon Checklist: Is it a reliable assessment of frailty? *Geriatrics & Gerontology International* 2016; 16: 893-902.

40. Kobayashi S, Murakami K, Sasaki S *et al.* Comparison of relative validity of food group intakes estimated by comprehensive and brief-type self-administered diet history questionnaires against 16 d dietary records in Japanese adults. *Public Health Nutrition* 2011; 14: 1200-1211.

41. Kobayashi S, Honda S, Murakami K *et al.* Both comprehensive and brief self-administered diet history questionnaires satisfactorily rank nutrient intakes in Japanese adults. *Journal of Epidemiology* 2012; 22: 151-159.

42. Ohkawara K, Oshima Y, Hikihara Y, Ishikawa-Takata K, Tabata I, Tanaka S. Real-time estimation of daily physical activity intensity by a triaxial accelerometer and a gravity-removal classification algorithm. *The British journal of Nutrition* 2011; 105: 1681-1691.

43. Cole RJ, Kripke DF, Gruen W, Mullaney DJ, Gillin JC. Automatic sleep/wake identification from wrist activity. *Sleep* 1992; 15: 461-469.

44. Troiano RP, Berrigan D, Dodd KW, Mâsse LC, Tilert T, McDowell M. Physical activity in the United States measured by accelerometer. *Medicine and Science in Sports and Exercise* 2008; 40: 181-188.

45. Doi Y, Minowa M, Uchiyama M *et al.* Psychometric assessment of subjective sleep quality using the Japanese version of the Pittsburgh Sleep Quality Index (PSQI-J) in psychiatric disordered and control subjects. *Psychiatry Research* 2000; 97: 165-172.

46. Doi Y, Minowa M, Okawa M, Uchiyama M. Development of the Japanese version of the Pittsburgh Sleep Quality Index. *Japan Journal of Psychiatry Treatment* 1998; 13: 755-763 (in Japanese).

47. Osuka Y, Kojima N, Wakaba K, Miyauchi D, Tanaka K, Kim H. Effects of resistance training and/or beta-hydroxy-beta-methylbutyrate supplementation on muscle mass, muscle strength and physical performance in older women with reduced muscle mass: protocol for a randomised, double-blind, placebo-controlled trial. *BMJ Open* 2019; 9: e025723.
